# Graham‐Little Piccardi Lassueur syndrome and review of the literature

**DOI:** 10.1002/ccr3.4761

**Published:** 2021-09-05

**Authors:** Fares A. Alkhayal, Fahad Alsudairy, luluah al Mubarak, Hind M. Almohanna

**Affiliations:** ^1^ Dermatology and Dermatologic Surgery Department Prince Sultan Military Medical City Riyadh Saudi Arabia

**Keywords:** Graham‐Little Piccardi Lassueur syndrome, Lichen planopilaris, lichen planus, scaring alopecia

## Abstract

Graham‐Little Piccardi Lassueur Syndrome (GLPLS) is a rare variant of lichen planopilaris (LPP) which characterized by triad of fibrosing alopecia of the scalp, non‐fibrosing alopecia of the axilla and groin, and a follicular spinous papule over the body. LPP is a rare follicular subtype of lichen planus which causes scarring alopecia of scalp, and there are three clinical subtypes of LPP including classic lichen planopilaris, frontal fibrosing alopecia, and GLPLS. Herein, we describe an adult dark‐skinned Saudi male with GLPLS who has numerous body follicular papules, complete loss of axillary hair, and partial loss of groin hair in addition to patchy fibrosing alopecia of the scalp. To the best of our knowledge, this is the first reported case of GLPLS in Saudi Arabia.

## Graphical abstract

Graham‐Little Piccardi Lassueur Syndrome (GLPLS) is a rare variant of Lichen planopilaris(LPP) which characterized by triad of fibrosing alopecia of the scalp, non‐fibrosing alopecia of the axilla and groin, and a follicular spinous papule over body. LPP is a rare follicular subtype of lichen planus. Which affects female most commonly.

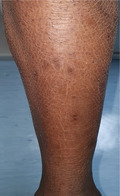

## 1 INTRODUCTION

Graham‐Little Piccardi Lassueur Syndrome (GLPLS) is a rare variant of lichen planopilaris (LPP) characterized by the triad of patchy scarring alopecia of the scalp, non‐scarring alopecia of the axilla and groin, and a numerous follicular keratotic papules over the body.^1^ It is more common in old age and in postmenopausal females, with only a very few reported cases in the literature wherein the disease has affected males in younger age.^2^ In our case, we report a young male who presented with features of cicatricial alopecia of the scalp, non‐cicatricial alopecia of the pubic region and axilla, and a follicular spinous papule over the body.

## 2 CASE REPORT

**FIGURE 1:**
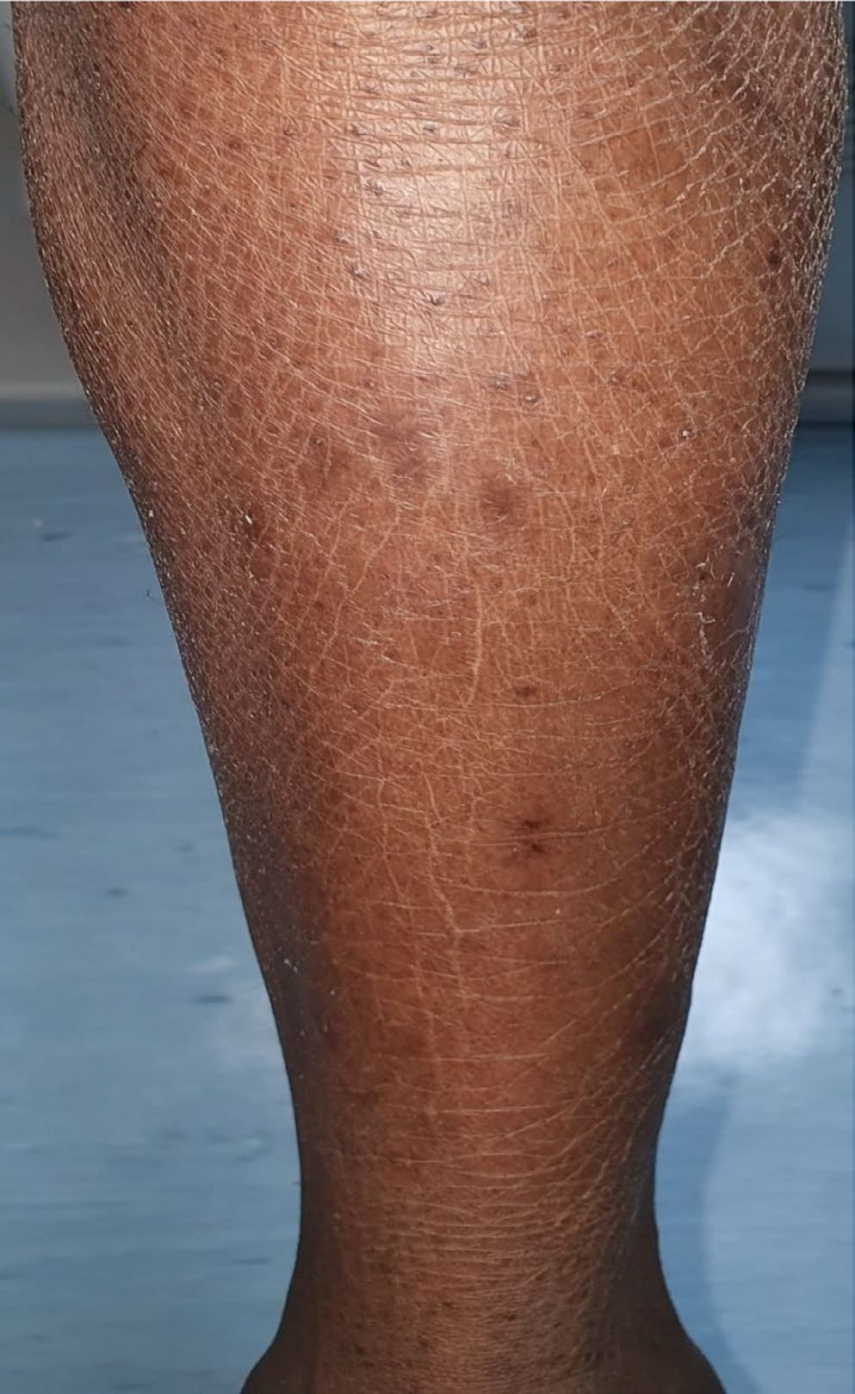
ichthyosiform scales, follicular papules, and hair loss involving right leg

**FIGURE 2:**
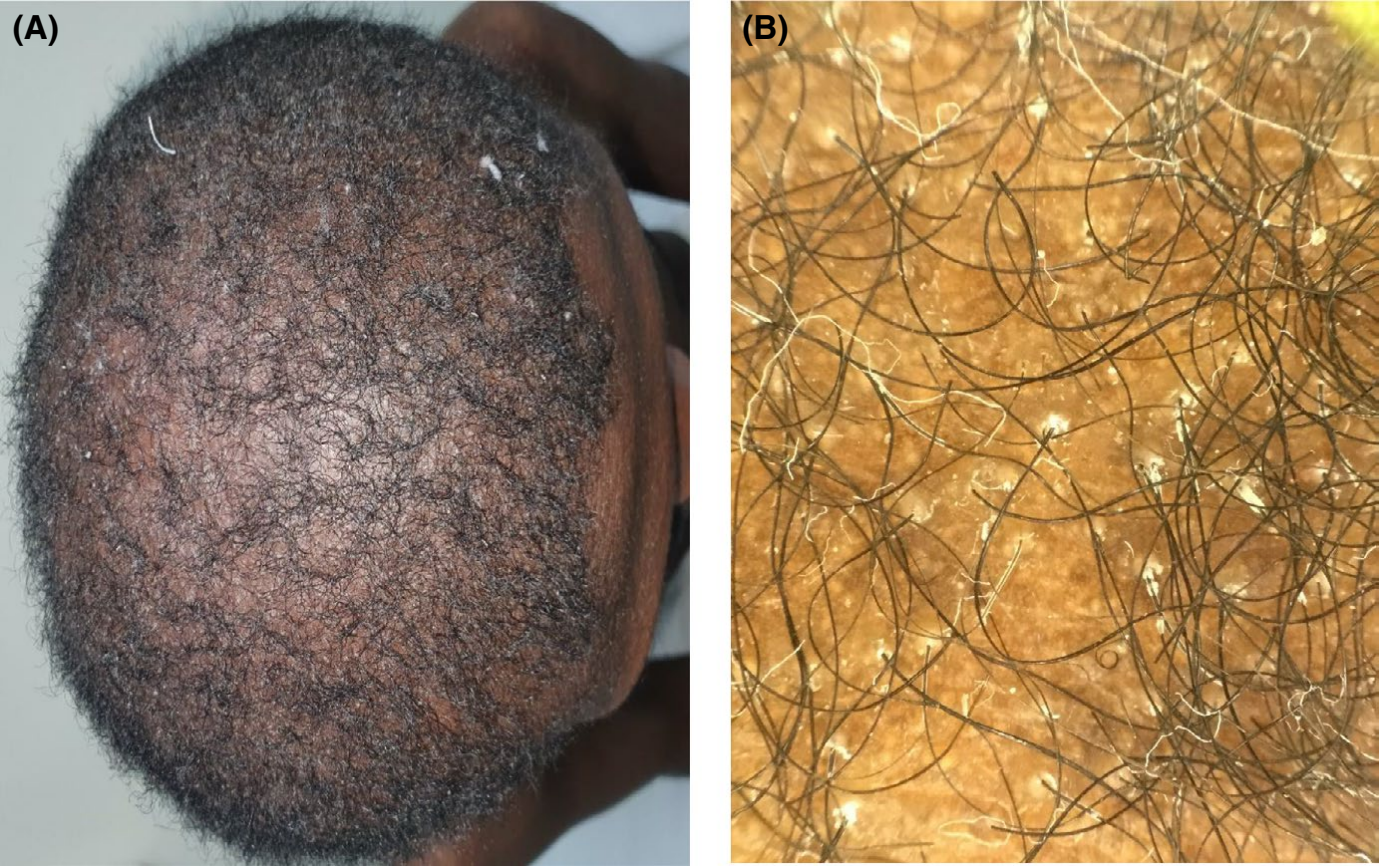
(A) diffuse scarring alopecia involving the scalp (B) dermatoscope shows peripilar cast with loss of follicular openings

**FIGURE 3:**
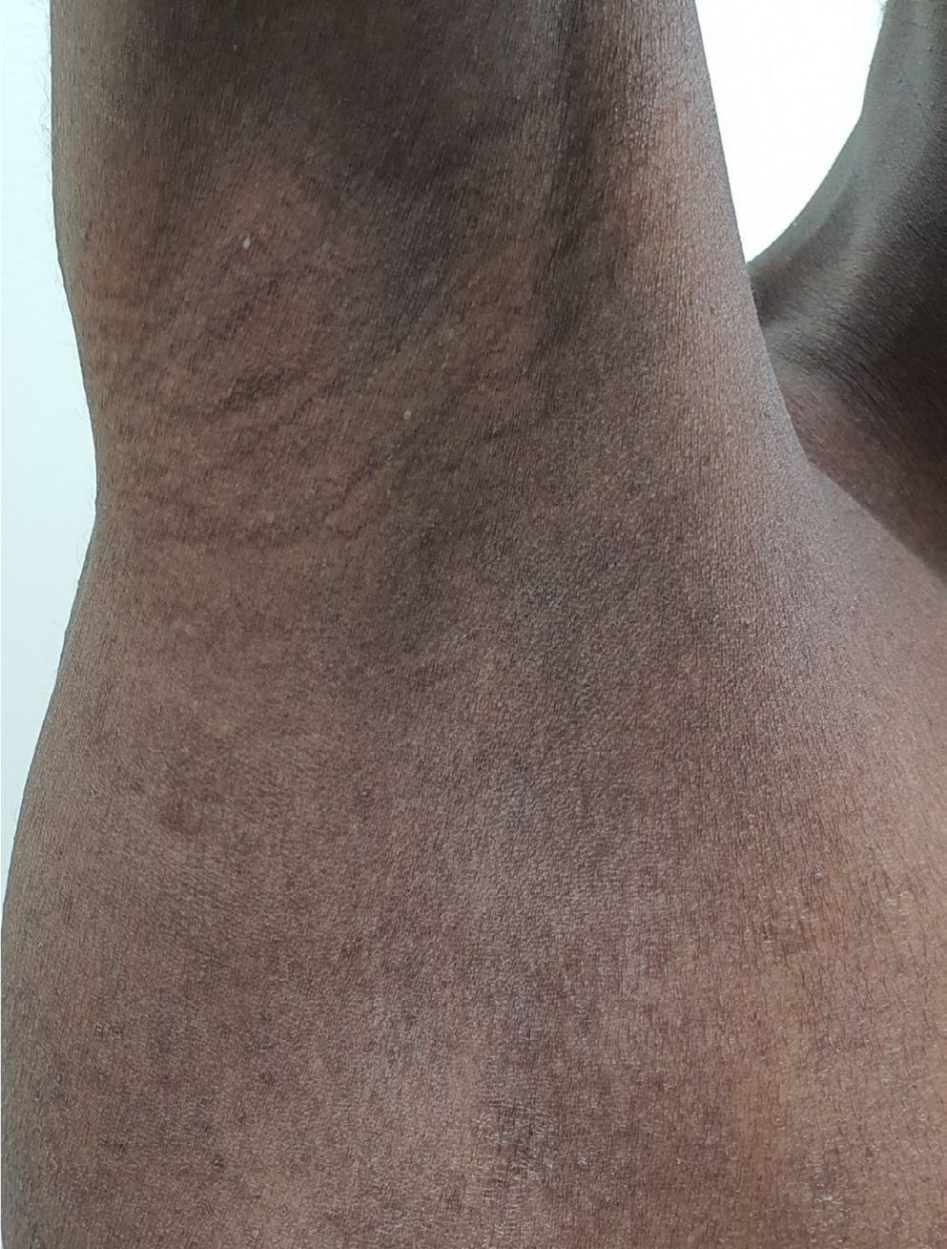
Non‐scarring alopecia of right axilla also shows scattered follicular keratotic papules on the trunk which is seen in GLPL syndrome

**FIGURE 4:**
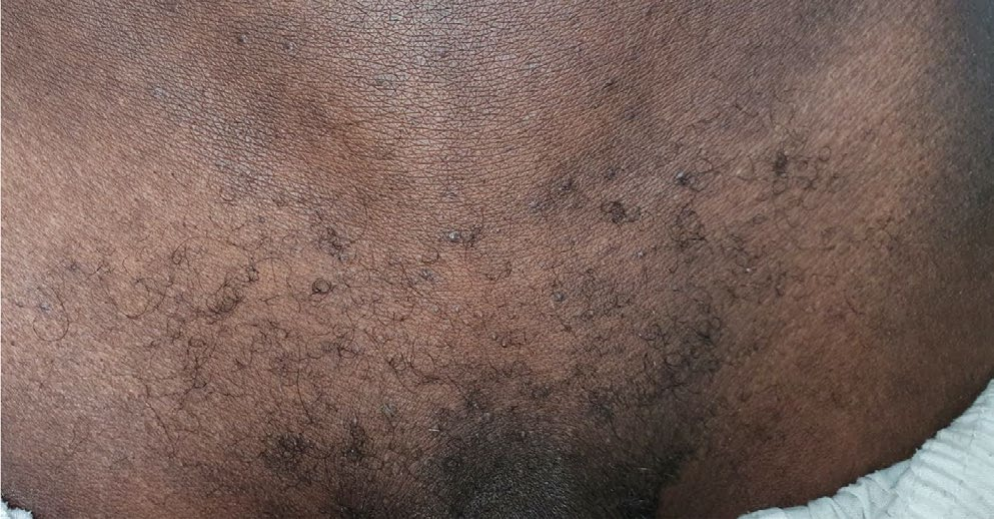
Patchy alopecia with follicular papules involving pubic hairs

**FIGURE 5:**
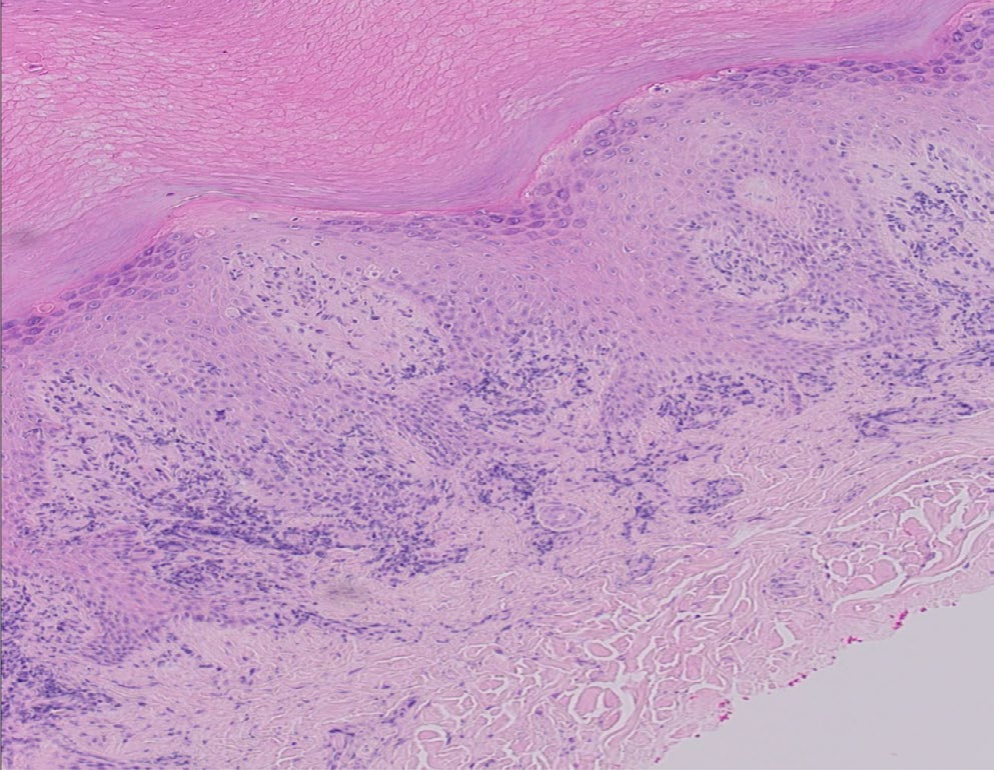
Microscopic examination of a biopsy taken from follicular papule of right leg showed orthokeratosis, hypergranulosis, acanthosis, and lichenoid infiltrate with necrotic keratinocytes (stained with hematoxylin and eosin stain H&E)

A 26‐year‐old healthy dark‐skinned male presented to our dermatology department complaining of severe pruritus all over body including the scalp for more than 3 years and progressive hair loss. Family history was negative for a similar condition. On examination, the patient's skin was xerotic with ichthyosiform scales over the extremities and numerous follicular papules all over the body (Figure 1). There was diffuse scarring alopecia involving the scalp (Figure 2A). Moreover, there was patchy alopecia involving the eyebrows, beard, and limbs hairs. The axillary hairs were lost (Figure 3) and there was patchy alopecia with follicular papules involving pubic hairs (Figure 4). Trichoscopic examination showed peripilar casts over the scalp (Figure 2B), eyebrows, beard, limbs hairs, and loss of follicular openings over scalp. Skin biopsy was taken from follicular papule over right leg showed orthokeratosis, hypergranulosis, acanthosis, and lichenoid infiltrate with necrotic keratinocytes (Figure 5). Mucous membrane examination was unremarkable, and patient has no family history of similar presentation. Based on the clinical and histopathological features, a diagnosis of (GLPLS) was made.

## 3 DISCUSSION

Graham‐Little‐Piccardi‐Lassueur syndrome GLPLS was first described by Piccardi in 1913. A second case was then described by Graham‐Little in 1915 in a patient referred by Lassueur, resulting in the name it bears today.^3^ Around 50 cases of GLPLS have been reported since then.^4^ The condition presents most commonly in middle‐aged white women and is characterized by a triad of cicatricial alopecia of the scalp, non‐scarring alopecia of the axillae and/or groin, and a follicular papule over body. Its cause remains unknown, but more likely is a T cell–mediated autoimmune condition.^4^ Recent studies showed that there is decrease expression of peroxisome proliferator‐activated receptor (PPAR) and many patients respond well to PPARγ agonists.^5^ Also, interferon and JAK singling are upregulated in LPP.^6^

The goal of treatment in GLPLS as well as in other scarring alopecia is to prevent progression of hair loss; thus, early diagnosis and intervention are crucial.^1^ Many treatment modalities have been used in treating lichen planopilaris with variable results. Treatment options range from topical and intralesional steroid to systemic treatment such as hydroxychloroquine, cyclosporine, and pioglitazone.^7^ Baibergenova and Walsh^8^ used PPARγ agonists (Pioglitazone) which induced complete remission in 25% and significantly improved symptoms in 50% of patient diagnosed to have LPP. Pioglitazone side effects are very mild including calf pain, lightheadedness, nausea, dizziness, and hives which were experienced by less than 5% of patients.^8^ Chiang et al.^9^ studied the use of hydroxychloroquine in the treatment of LPP in 40 patients for 12 months. Their results showed that hydroxychloroquine was very effective in terms of controlling symptoms and halting disease progression with a 69% and 83% significant reduction in severity of LPP at both 6 and 12 months, respectively. Treatment with oral tofacitinib either as monotherapy or as adjuvant to other treatment showed measurable 80% improvement clinically.^6^ Excimer laser (308‐nm) was used by Navarini et al. twice weekly in 13 patients and all patient experienced relief of pruritus with 40% reduction in inflammation but only 25% of patients had hair regrowth.^10^ Finally, naltrexone was used and showed improvement mainly in term of relieving symptoms such as pruritus.^11^ Our patient was started on hydroxychloroquine after he was evaluated by ophthalmology, and there was no contraindication to start the medication. In addition, the patient was started on topical treatment in the form of tretinoin 0.05% cream targeting follicular keratotic papules. On follow‐up, the patient reported improvement in term of pruritis and reduction on the severity of follicular keratotic papules.

## 4 CONCLUSION

GLPLS is a rare variant of LPP characterized by the triad of patchy fibrosing alopecia of the scalp, non‐fibrosing alopecia of the axilla and groin, and a follicular spinous papule on the body. The exact cause still unknown and the goal of treatment is to stop disease progression and to reduce associated symptoms.

## CONFLICTS OF INTEREST

The authors report no conflicts of interest.

## AUTHOR CONTRIBUTIONS

Dr. Alkhayal was first one to examine the patient and doing biopsy and he wrote the introduction and abstract in addition to literature review. Dr. Hind took the photograph with dermtascope and she did Literature review plus auditing the case. Dr almubark and alsudairy wrote the conclusion and review the case.

## ETHICAL APPROVAL

Patient‐informed consent was signed by the patient.

## Data Availability

The data that support the findings of this study are available without restriction. All data are included within the manuscript.
